# A Comparative Study on the Texture of Exposed Aggregate Concrete (EAC) Pavements Using Different Measurement Techniques

**DOI:** 10.3390/ma17164147

**Published:** 2024-08-22

**Authors:** Pawel Gierasimiuk, Marta Wasilewska, Wladyslaw Gardziejczyk

**Affiliations:** Faculty of Civil Engineering and Environmental Sciences, Bialystok University of Technology, Wiejska 45E Street, 15-351 Bialystok, Poland; marta.wasilewska@pb.edu.pl (M.W.); w.gardziejczyk@pb.edu.pl (W.G.)

**Keywords:** exposed aggregate concrete, macrotexture, microtexture, mean profile depth, mean texture depth, friction coefficient

## Abstract

This paper presents issues related to the assessment of the texture of aggregate concrete (EAC) surfaces using various methods for its verification. Microtexture was assessed using the British Pendulum Tester (BPT) and Dynamic Friction Tester (DFT). Two laser profilometers were used to assess macrotexture, circular texture meter (CTM) and stationary laser profilograph (SPL), as well as the commonly known volumetric method. Measurements were carried out on left and right tracks and in between them on five test sections of expressways. Based on the analyses performed, it was found that the results obtained by the DFT were less sensitive to changes in microtexture between individual tracks compared to the results obtained by the BPT. The BPN values in the left track were lower than those in the right track. However, the difference between the DFT20 results in these spots was insignificant. Both MPD and MTD values did not show significant differences between the right and left tracks. However, some differences were observed between the MPD parameters obtained using the CTM and SPL. This resulted from the different frequency and length of the scanned surface profile. However, the differences were at an acceptable level. A very high linear correlation was obtained in the case of BPN and DFT20 values (r − 0.719), and in the case of MPD and MTD values, the correlation was almost certain (r above 0.900). Based on a comparative analysis of the models estimating mean texture depth (MTD/ETD), a significant difference was observed between models based on EAC pavement results and those based on asphalt surfaces.

## 1. Introduction

The texture of a pavement’s upper layer has a significant impact on the value of the friction force between the tire and the road surface, which plays a key role in both the dynamics and kinematic reactions of vehicles, as well as the stability and maneuverability of a vehicle in critical driving conditions. According to the classification system contained in [1,2], texture is divided into megatexture, macrotexture and microtexture, according to the wavelength and the amplitude. Megatexture is unevenness corresponding to wavelengths above 50 mm, which adversely affect driving comfort, rolling resistance, interior noise and road user safety. They are caused by damage to the pavement, i.e., cracks, defects, etc. The macrotexture corresponds to unevenness with a wavelength from 0.5 mm to 50 mm. It affects not only noise but also the drainage of water from contact between the tire and the road pavement, thus reducing the risk of aquaplaning [3,4]. The level of this unevenness results, in the case of concrete pavements, in the texturing method [5,6]. Microtexture is characterized by unevenness with a wavelength of less than 0.5 mm. It is responsible for breaking the water film to create a dry surface to generate the required friction force. It depends primarily on the polishing resistance of the coarse aggregate and the content of fine aggregate in the wearing course [7,8,9]. Due to the role of texture in ensuring the appropriate level of skid resistance, in many countries, it is mandatory to check it during the period of use of the pavement.

In practice, texture in terms of macrotexture unevenness is assessed using the volumetric method based on the MTD (Mean Texture Depth) parameter determined in accordance with EN 13036-1 [10] or the profilometric method based on the MPD (Mean Profile Depth) parameter in accordance with EN ISO 13473-1 [11]. Both methods are very well described in the literature [12,13]. Although the volumetric method is a very cheap and easy solution for obtaining information about texture, it does not enable a continuous registration of changes on the tested surface. Additionally, the measurement requires time and disabling the tested section from traffic. The development of contactless pavement scanning techniques made it possible to replace the volumetric method with parameters based on profile registration. The parameters determined on their basis may not only be MPD but also Ra (Arithmetic mean deviation), Rms (Root Mean Square), Rsk (Skewness) and Rku (Kurtosis). Many stationary and dynamic laser profilometers have been developed, and they must meet the requirements of the standard EN ISO 13473-1. Stationary devices enable measurements both in laboratory and in situ. However, similarly to the volumetric method, these measurements require road closure at some point. Dynamic profilometers are mounted on a vehicle; therefore, they have better performance compared to stationary profilometers. This enables continuous data recording. However, they cannot perform measurements in difficult-to-reach places, e.g., roundabouts, intersections, road junctions or during road construction. It is thus recommended to use stationary profilometers [14,15].

The registration of microtexture unevenness requires specialized profilometers. For this purpose, scanning images taken under a microscope or 3D scanners are primarily used [16]. Images of the upper layer surface of the pavement are also used to assess the macrotexture, and the development of technology in recent years makes it also possible to determine the parameters characterizing the pavement texture in 3D format [17,18,19,20]. Using such equipment in road conditions is very difficult or even impossible. Therefore, studies on the assessment of microtexture are carried out in the laboratory on samples cut from the pavement or samples prepared in the laboratory and simulated phenomena occurring in real traffic conditions. However, an indirect measure for assessing microtexture in situ are the results obtained from stationary or dynamic devices simulating braking conditions with full wheel lock at low slip speed (10–20 kph). It should be noted that the advantage of stationary devices for both macrotexture and microtexture assessment is the ability to use calibration plates to check the reliability of the obtained results. In the case of dynamic devices, quality control procedures are more complex.

Parameters describing the texture on both the macro and micro scale are taken into account in numerous models that have been developed to predict parameters related to the friction generated at the tire–pavement interface [21]. Some researchers emphasize that the division of the texture scale presented in EN ISO 13473-1 [11] is simplified and has limitations [22,23]. The use of decomposition methods, i.e., wavelet transform, Hilbert–Huang transform or Fourier transform, to identify individual scales showed that simplified division between microtexture and macrotexture does not represent their complex impact on the level of friction force values. An alternative approach to describing the texture is to analyze pavement unevenness through parameters closely related to the height, shape and density of the irregularities. However, it has been proven that the characterization of textures largely depends on the resolution of the recorded surface topography and specialized measurement techniques [24]. This study makes a significant contribution to understanding the mechanisms related to texture and its impact on the phenomena occurring at the interface between the tire and the road pavement. In practice, dynamic or stationary laser profilometers and devices reflecting full wheel lock braking are used to monitor texture.

Texture is closely related to the technology of construction of the pavement’s upper layer. Consequently, this means that the proper selection of materials, determination of the composition of mixtures, texturing techniques, etc. will allow for maintaining the required level of skid resistance during the pavement life cycle.

EAC pavements are not as popular a technology used in road construction as asphalt pavements. This type of pavement is primarily used for roads with heavy traffic—mainly expressways and highways. Properly selected proportions between individual aggregate fractions, the maximum size of aggregate grains, and the texturing process allow for the arrangement of coarse aggregate to be such that a positive texture profile is created [25,26,27]. It should be noted that EAC pavements are characterized by a very good durability of the macrotexture during road use, unlike concrete pavements textured using burlap drag, brushing or grooving of fresh concrete mix [28,29]. It should be noted that EAC is a very difficult technology for constructing upper layers of road pavements. One of the difficulties in the construction of EAC pavements is the requirement to obtain a homogeneous texture [3]. This is due to the difficulty in determining the appropriate time from the moment of spraying the compacted concrete mixture with a set-retarding agent to the beginning of the removal of unbound cement mortar and exposure of the aggregate, taking into account numerous environmental factors such as wind velocity, air temperature and humidity [30]. To obtain the proper texture, it is very important to comply with the regimes during its construction, especially at the stage of its texturing and curing. If a mistake is made, repair is very time-consuming and expensive compared to layers of asphalt mixtures. Therefore, controlling texture conditions is very important because it affects not only skid resistance but also rolling resistance and tire/road noise [5,31,32].

Some researchers make use of a given texture assessment method depending on the type of road pavement. For example, assessing the texture of open (PA) or semi-open (SMA LA, BBTM) pavements is debatable. Profilometric methods do not accurately reflect the shape of the surface irregularities of free spaces due to open texture. However, in the case of the volumetric method, it is possible that the glass beads or sand used for the test penetrate into the free spaces of the layer structure, thus not reflecting the texture surface. In such cases, determining the relationship between profilometric and volumetric methods is inadvisable [33]. In the case of concrete pavements, when choosing a method for assessing texture, attention should be paid to the surface texturing technique. On surfaces textured with burlap drag or brush, spreading glass beads or calibrated sand is very difficult due to the low texture depth (below 0.5 mm). Therefore, it is recommended to use profilometric methods. However, in the case of longitudinally grooved concrete pavements, when using a dynamic laser profilometer recording a 2D profile, there is a risk of underestimating the depth of the pavement texture. The laser beam can record a profile in the groove, i.e., a flat surface. Therefore, it is recommended to use a 3D laser that will fully reflect pavement irregularities. However, when using stationary devices, they must be properly positioned on the pavement during measurement. The texture of EAC pavements can be assessed by both profilometric and volumetric methods, as they are multi-directional. There are numerous regression models available in the literature that were developed on the basis of results obtained using volumetric and profilometric methods using both dynamic and stationary devices [17,19,34,35,36]. These models are dedicated to estimating parameters describing the texture or friction coefficient. They were mainly developed based on measurements of asphalt mixture layers. This is due to the fact that this technology for pavements’ upper layers is the most frequently used in the world and very well described in the technical literature. Concrete pavements are an alternative to building roads using asphalt mixtures. They are mostly preferred for roads with very heavy traffic.

The aim of this article is to provide a comparative analysis of parameters describing texture using various stationary devices, in terms of assessing Exposed Aggregate Concrete pavement in real conditions on the road. This study includes an analysis of texture variability in the cross-section of the road lane, taking into account the left, right and center tracks. Additionally, the verification of regression models developed by other researchers for estimating parameters describing texture was presented, using the results of this study recorded in actual road conditions.

## 2. Research Program

**Figure 1:**
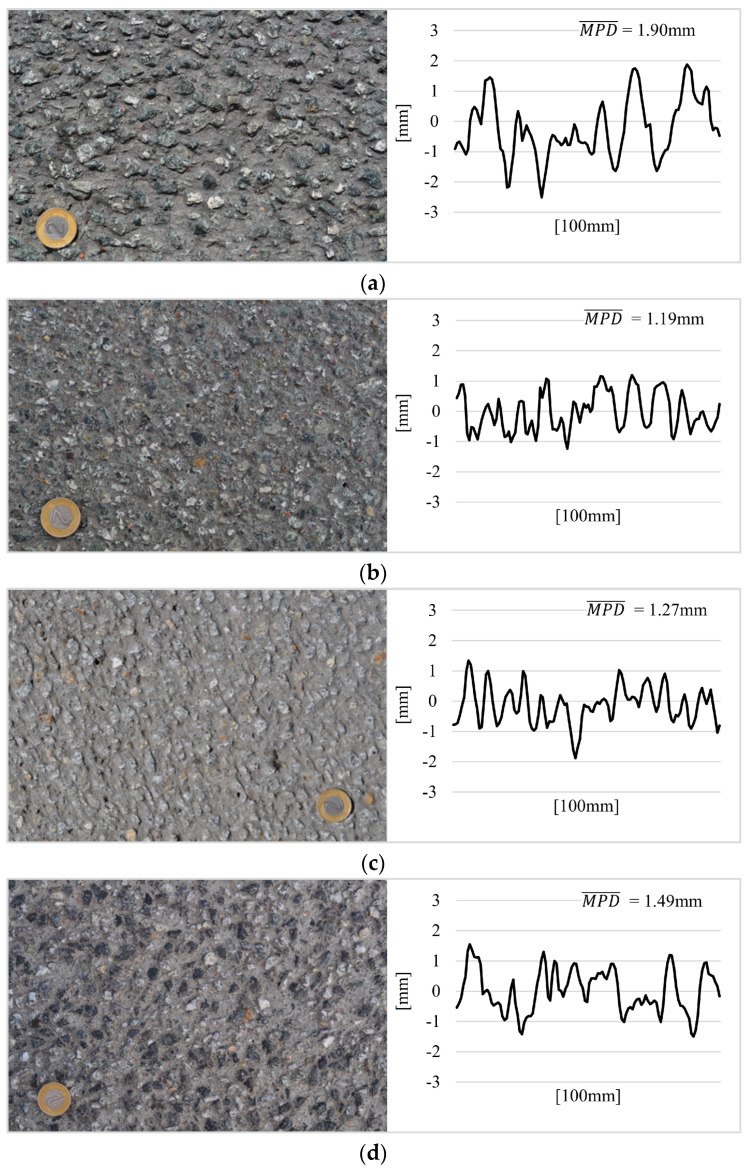

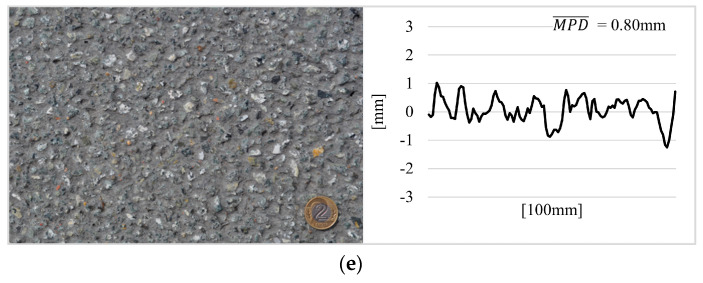
Pavements of test sections with samples of unevenness profiles (the coin has a diameter of 21.5 mm): (**a**) TS−1, (**b**) TS−2, (**c**) TS−3, (**d**) TS−4 and (**e**) TS−5.

Five test EAC sections (TS−1, TS−2, TS−3, TS−4, TS−5; TS−Test Section) with a length of 100.0 m located on expressways were selected for testing. The test sections were selected on the basis of preliminary measurements of the MPD parameter, which were used to determine its impact on road surface noise presented in the article [5]. Sections were selected that varied in texture level. Figure 1 shows images of the tested pavements and samples of their unevenness profiles.

The scope of this research included the following measurements:

– British Pendulum Number (BPN) using the British Pendulum Tester (BPT) according to ASTM 303 [37];
– Friction coefficient DFT20 using Dynamic Friction Tester (DFT) according to ASTM E1911 [38];
– MPD values using Circular Texture Meter (CTM) according to ASTM E2157-01 [39];
– MPD values using Stationary Laser Profilograph (SLP) according to EN ISO 13473-1 [11];
– MTD values according to EN 13036-1 [10].

**Figure 2:**
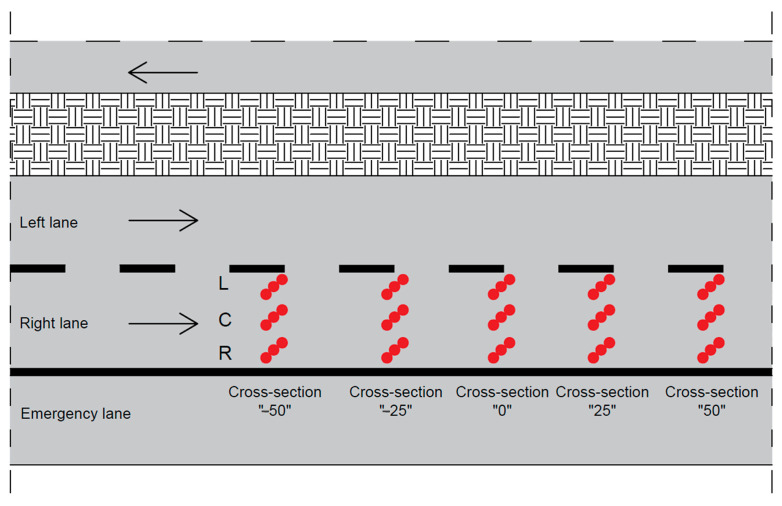
Location of measurement cross-sections and test spots on the test section.

Five measurement cross-sections were marked every 25.0 m on the right lane of the road on each test section. Measurements were carried out in the left wheel track (L—left), in the right wheel track (R—right) and between the wheel tracks (C—center) (Figure 1). Three measurements of each parameter were made in each track (Figure 2). The total number of test spots on the section was 45.

**Figure 3:**
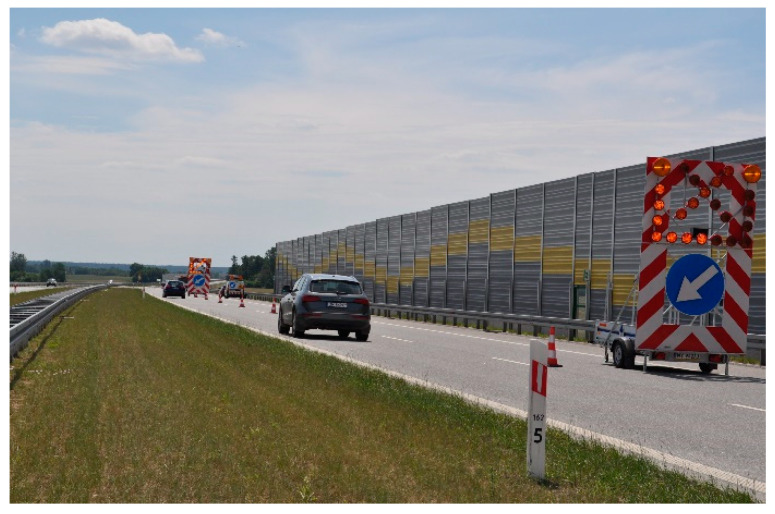
Example of a test section.

The test sections were marked on the expressway. In order to ensure optimal safety during the measurements, the right lane of the road was closed for approximately 500 m in each section (Figure 3).

## 3. Description of Measurement Procedures

### 3.1. Measurement of the BPN

**Figure 4:**
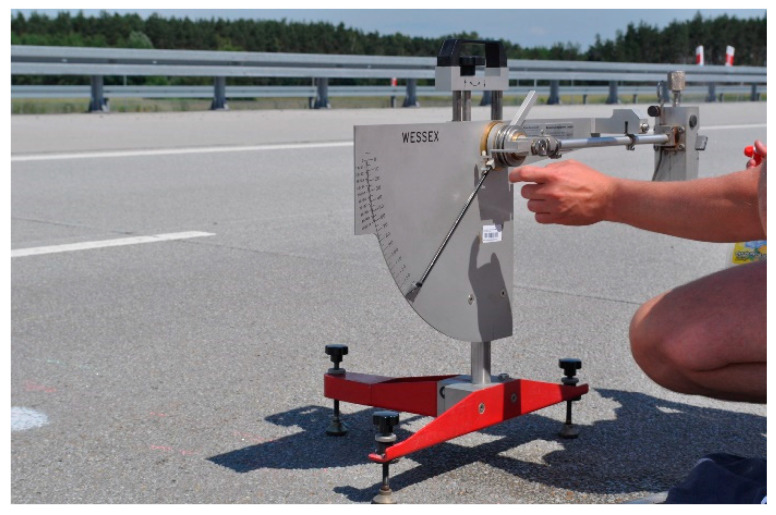
Measurement of British Pendulum Tester.

The BPN value was determined by measuring the friction between the rubber slider of the British Pendulum Tester and the pavement (Figure 4). Before the measurement, the pendulum was calibrated and then adjusted so that the length of the slide was 126 mm. The surface and the slider of the pendulum were then wetted with water. The pendulum arm with the slider was released, and the friction value was read from the large scale. This operation was repeated 5 times. The BPN value was calculated from the last three readings.

### 3.2. Measurement of the DFT20

**Figure 5:**
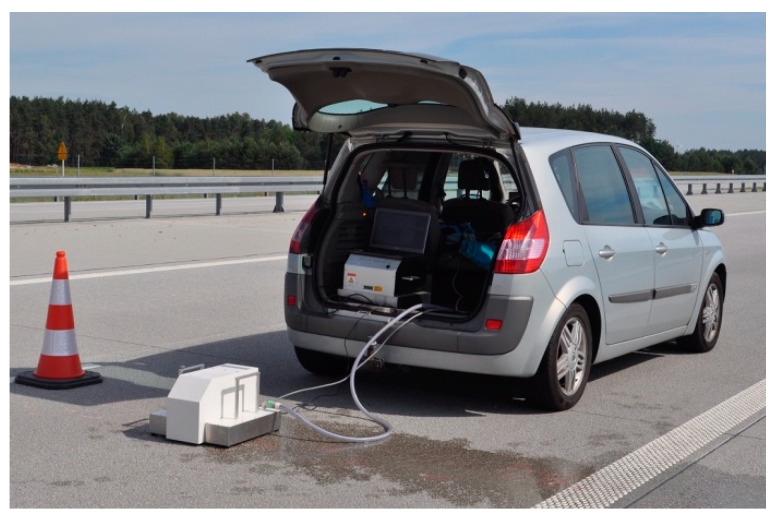
DFT device during field measurements.

The Dynamic Friction Tester is a stationary device in which the friction coefficient is measured in the slip speed ranging from 0 kph to 80 kph (Figure 5). The measurement principles correspond to mobile locked wheel devices. The DFT consists of two disks that are connected to each other by a spring and a sensor. There are three rubber sliders mounted on the lower disk. Discs rotating at 80 kph are lowered to the pavement, and the friction coefficient is measured. The software allows for determining the friction coefficient DFT20 at a slip speed of 20 kph, DFT40 at 40 kph and DFT60 at 60 kph. The DFT20 parameter was adopted for the analysis.

### 3.3. Measurement of the MTD Using Volumetric Patch Method (VPM)

**Figure 6:**
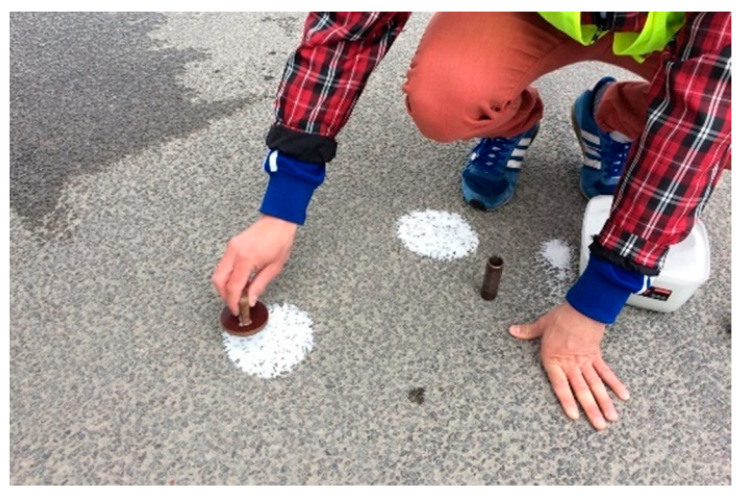
Macrotexture measurements using the volumetric patch method.

Measurement involves spreading glass beads with dimensions of 180/250 μm and a volume of 25,000 mm^3^ with a rubber disk so that they form a circle on the tested pavement (Figure 6). This operation was repeated in four places in the measurement area. The average texture depth—MTD (Mean Texture Depth)—was calculated based on the measured circle diameter.

### 3.4. Measurement of the MPD Using the CTM

**Figure 7:**
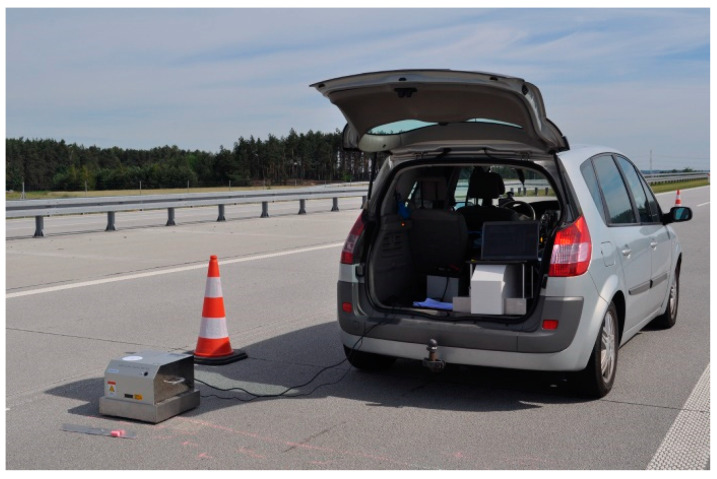
CTM during measurements.

The Circular Texture Meter (CTM) (Figure 7) determines the unevenness profile based on which parameters characterizing the macrotexture are calculated—MPD and RMS. The profile is measured by a CCD laser displacement sensor that moves around the circumference of a circle with a radius of 142 mm. The resolution of the device is 0.87 mm. The MPD_CTM_ parameter was adopted for the analysis. Before the measurement, the correct operation of the sensor was checked on a calibration board with a known profile.

### 3.5. Measurement of the MPD Using Stationary Laser Profilometer (SLP)

**Figure 8:**
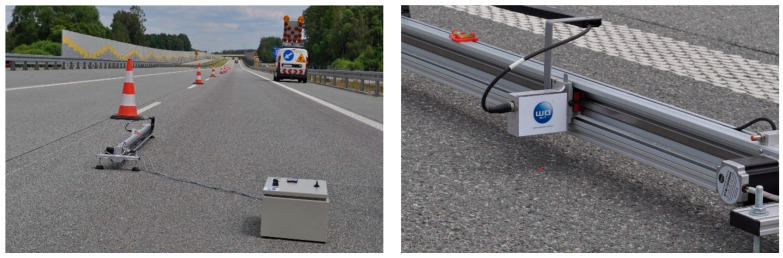
Stationary Laser Profilometer.

The SLP device was built at the Bialystok University of Technology in Bialystok (Figure 8). The measuring part is 1.7 m long. The SLP was equipped with a laser sensor with parameters similar to the laser sensor from the CTM device. As a result of the measurement, a surface texture profile was obtained; based on this, the MPD value was determined. The sampling resolution could be 0.86 mm or 0.43 mm. The MPD_SPL_ results obtained at a resolution of 0.43 mm were taken into account for the analysis.

## 4. Results and Discussion

### 4.1. Microtexture Assessment Using a Method Based on DFT and BPT Measurements

**Figure 9:**
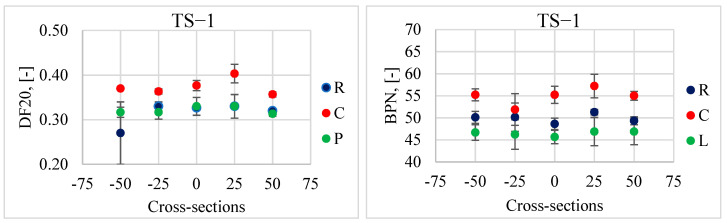

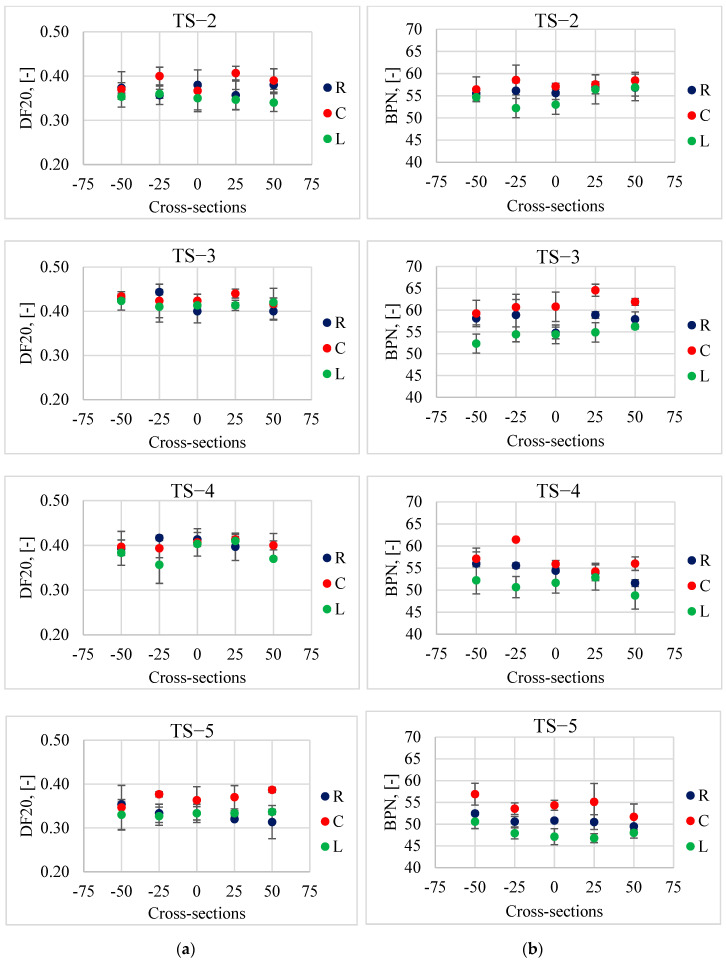
Average values of DFT20 (**a**) and BPN (**b**) with standard deviations on individual test sections.

Figure 9 shows the results of the DFT20 and BPN friction coefficients in the left (L), and right (R) track and between the wheel tracks (C) on individual test sections. Table 1 summarizes their descriptive statistics, i.e., average values. STD represents standard deviations, and V represents coefficients of variation.

The difference between the DFT20 results recorded in individual tracks is only noticeable on sections TS-1 and TS-5. In the remaining sections, the differences are insignificant. The DFT device is clearly less sensitive to microtexture variations compared to the British Pendulum Tester. This is evidenced by slight differences in the DFT20 values between the left and right track. The most polished part of the lane is the left traffic track. This is due to the higher frequency and higher speed of crossings resulting from overtaking maneuvers. This is clearly confirmed by the results of tests conducted by the BPT on the EAC pavements. There is a significant difference between the BPN values depending on the location of the analyzed tracks on the tested sections; the highest values were obtained in the middle (C), then in the right (R) and left track (L).

The coefficients of variation indicate that the surfaces are homogeneous in terms of microtexture in individual tracks (C, L, R). The values are in a range of 3 ÷ 8%. A larger scatter was only recorded when measured with the DFT device on the TS-1 section in the right track.

### 4.2. Macrotexture Assessment by Volumetric and Profilometric Methods

**Figure 10:**
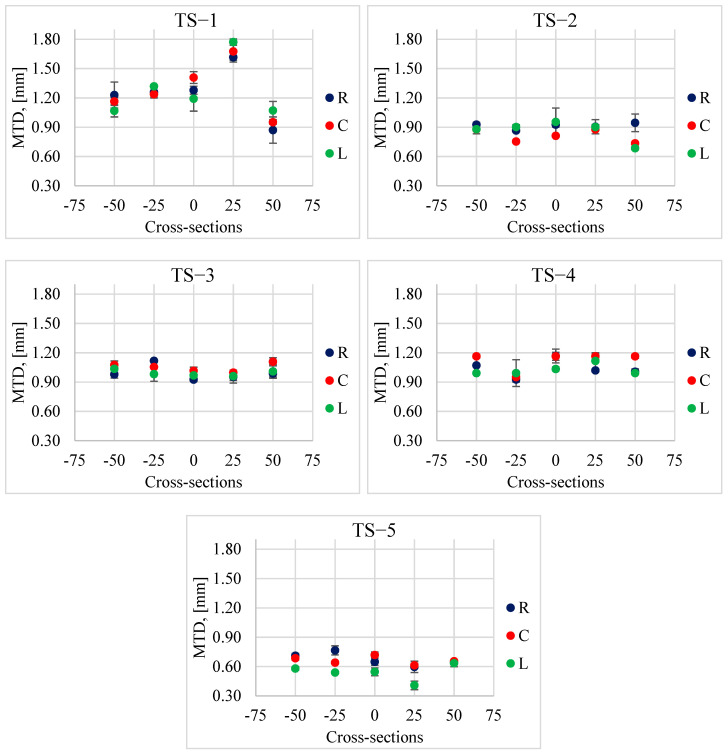
Average MTD values with standard deviations on individual test sections.

**Figure 11:**
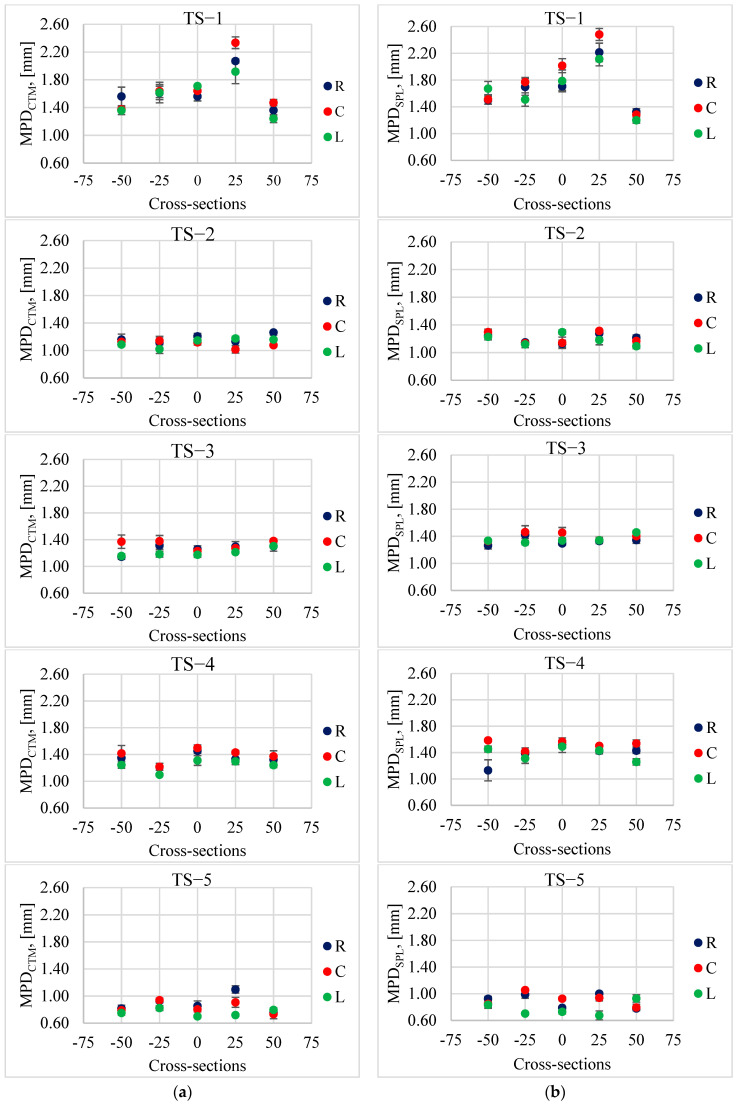
Average MPD values with standard deviations on individual test sections obtained from (**a**) CTM and (**b**) SPL.

The measurement results of the MPD and MTD parameters recorded on individual test sections in the left wheel track (L), in the right wheel track (R) and between the wheel tracks (C) are presented in Figure 10 and Figure 11. Table 2 provides descriptive statistics, i.e., average values, standard deviations (STD) and coefficients of variation (V).

In some rare cases, slightly lower MPD and MTD values were observed in the left track compared to the values obtained in the right and the middle tracks of the lane. Generally, it was found that the differences in the parameters describing the macrotexture depending on the track (L, R or C) were insignificant. This is the opposite situation compared to changes in the values of friction coefficients, BPN and DFT20, where the track was important. In the case of macrotexture changes, the recorded differences were dictated by the differences in the macrotexture level of the selected test sections. However, when analyzing changes on a given section, the differences were visible in the cross-sections and not in the track of the vehicle’s wheels. This may be related to the process of curing and exposing the coarse aggregate.

It was noticed that the results obtained from individual measurement techniques show the same trends in changes at individual measurement points on the test sections. This is especially visible on sections TS−1 and TS−4. In the case of TS−1, the highest values were obtained at locations “0” and “+25”, which result from the loss of aggregates from the surface. In these sections, high values of all analyzed parameters were obtained, MPD_CTM_, MPD_SLP_ and MTD.

**Figure 12:**
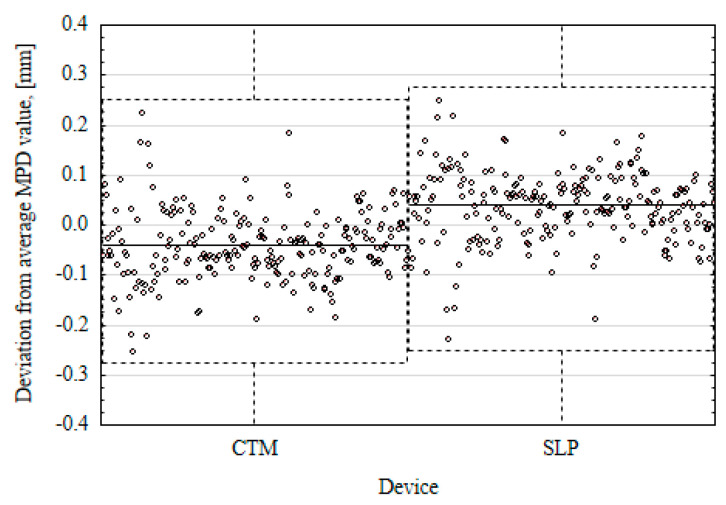
Deviation from the average MPD value for a particular profilometer.

When comparing the parameters obtained from volumetric and profilometric methods, it is evident that the MTD values are lower than MPD_CTM_ and MPD_SLP_. However, there are slight differences between the MPD results from CTM and SLP. The methods for determining the average profile depth in both cases meet the requirements specified in EN-ISO 13473-1 [11]. However, the CTM device scans the surface profile in a circle, while the SLP device scans it in a straight line. Therefore, it is not possible for each of these devices to scan the profile in exactly the same place on the tested surface. This causes differences in the results. Figure 12 shows the deviation from the average MPD (1.27 mm) calculated from a set of data at 225 measurement points located in individual cross-sections on test sections, which were obtained from measurements with two repetitions (runs) with CTM and SPL devices.

The range of deviations calculated at individual measurement points for two devices is comparable. Therefore, differences are accepted.

### 4.3. Determining Linear Regression between Individual Parameters

**Figure 13:**
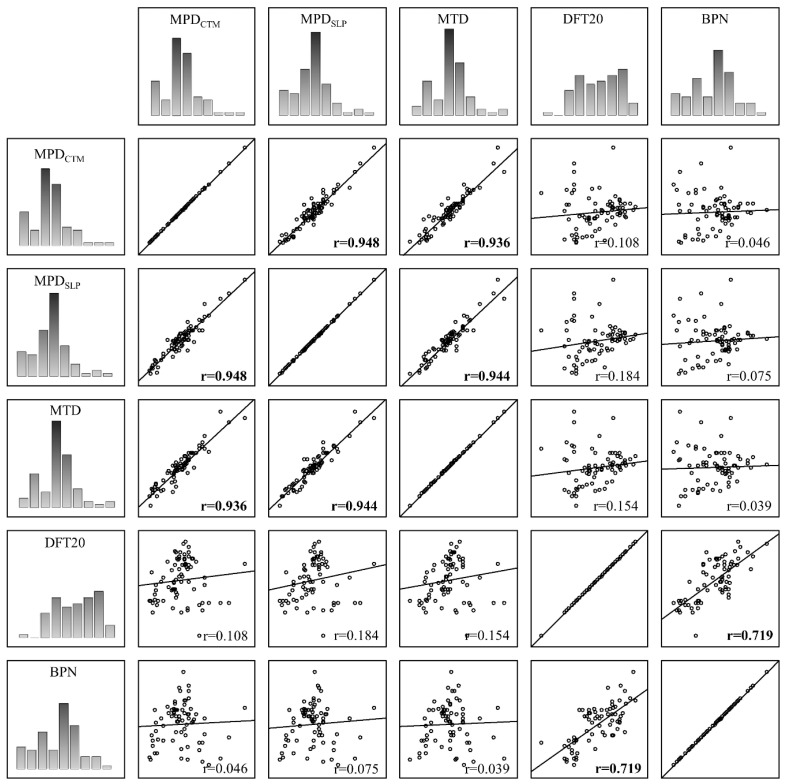
Matrix scatterplot.

A correlation analysis was performed to determine linear relationships between the analyzed parameters characterizing microtexture and macrotexture. The matrix scatterplot for each pair of variables obtained from microtexture and macrotexture and the linear regression lines are presented in Figure 13. Pearson correlation coefficients, r, are also shown in each scatter plot.

**Figure 14:**
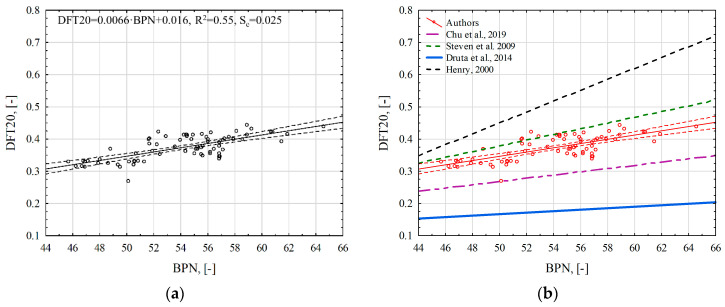
Regression relationships between DFT20 and BPN coefficients: (**a**) regression relationship with 95% confidence interval; (**b**) comparison of the regression relationship of the authors and other researchers [34,35,36,40].

It was found that there was no correlation between the parameters describing the microtexture (BPN, DFT20) and the parameters describing the macrotexture (MTD, MPD). This is also confirmed by the results obtained by other researchers [9,23]. However, a very high correlation was obtained in the case of BPN and DFT20 (r − 0.719), and, in the case of MPD and MTD, the correlation was almost full (r above 0.900). Therefore, linear regression relationships were determined in these configurations of variables (Figure 14).

The obtained model (Figure 14a) explains 55% of the DFT20 values through the values of the independent variable BPN. Table 3 lists the regression relationships established by individual researchers, and Figure 14b presents their graphical presentation. They were determined on the basis of measurements primarily made on wearing courses made from asphalt mixtures.

The relationships differ in the values of the regression coefficients b0 and b1 and the coefficient of determination R^2^. They may result from the number and spread of results that were the basis for developing these models. BPT and DFT devices reproduce braking conditions with a locked wheel during measurement. The analysis of the results proved that both DFT and BPT are sensitive to changes in microtexture. However, differences between devices related to sliding speed, slider size and rubber hardness most likely influence the differences in the mechanism of generating friction force between DFT and BPN. Consequently, the results obtained from these two devices cannot be used interchangeably.

**Figure 15:**
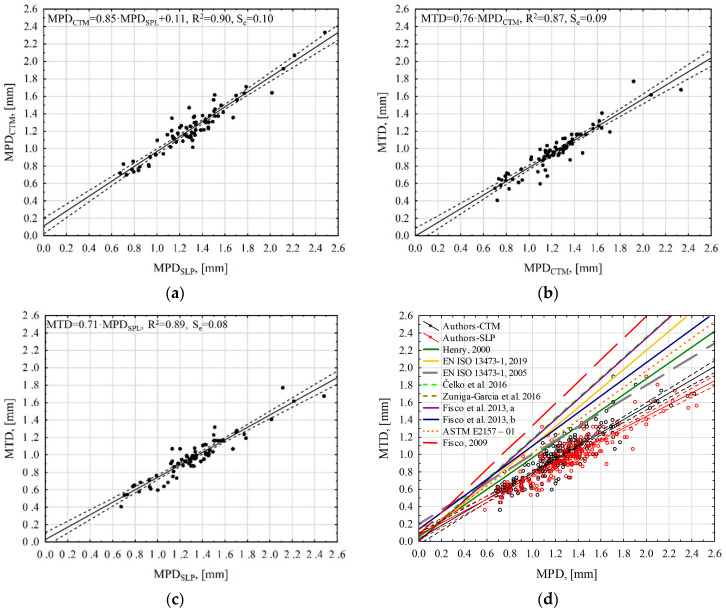
Regression relationships between macrotexture coefficients: (**a**) MPD_CTM_—MPD_SLP_, (**b**) MTD—MPD_CTM_, (**c**) MTD—MPD_SLP_ and (**d**) MTD—MPD comparison of the results of the authors and other researchers [11,15,17,19,20,34,39,41].

In the case of the relationship developed on the basis of the results of parameters describing the macrotexture profile, it was found that the MPD_SLP_ values explain approximately 90% of the variability of the MPD_CTM_ results (Figure 15a). Differences in surface profile scanning and laser sampling frequency contribute to some scatter in the results. However, statistical analysis has proven that these two devices can be used interchangeably when assessing macrotexture in a range from 0.40 mm to 2.00 mm, as the differences in results are approximately 10%. However, the relationships presented in Figure 15b,c, which were obtained using individual laser profilometers, explain 87% (MPD_CTM_) and 89% (MPD_SLP_) of the variability of the MTD parameter. Dynamic laser profilometers are usually used to diagnose the condition of the road surface. Dependencies that allow for MTD estimation based on MPD results or vice versa are practiced quite often. The reasons may be the desire to refer to archived data, hardware limitations or limitations in performing measurements with a specific device. The standard EN ISO 13473-1 [11], both the 2005 and 2019 versions, presents functional dependencies for estimating ETD (Estimated Texture Depth). However, many other linear relationships that use MTD and MPD results can be found in the technical literature. Table 4 shows dependencies, and Figure 15d shows their graphical presentation.

The values of the slope coefficients of the regression Equation (b1), determined by the authors, are much smaller than 1. However, in the remaining analyzed cases, most of the equations have a value around 1 or above this value. This may be due to the profile characteristics of the tested surface. To explain the differences, other parameters that can be determined from the profile should be analyzed. EAC pavements are characterized by a positive texture, and wearing courses made of asphalt mixtures, i.e., SMA, BBTM, PA, AC, are characterized by a negative texture. In the case of the analyzed EAC pavements, the Rsk value is close to or above 0, which indicates the positive nature of the texture (the Rsk parameter evaluates the symmetry of the texture curve with the baseline) [25,42].

**Figure 16:**
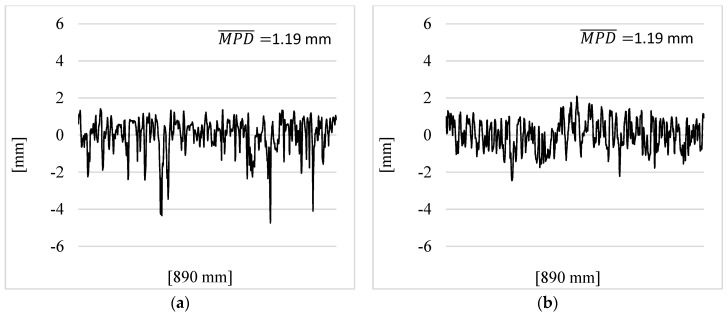
Examples of profiles obtained from measurement with the CTM device: (**a**) SMA11; (**b**) EAC8.

Due to the positive texture of the EAC, during macrotexture measurements using the volumetric method, glass beads are distributed in the spaces between the grains in a different way than on layers of asphalt mixtures with a negative profile. There is also a difference when calculating the MPD parameter. The average value of the profile height is much lower than in the case of negatively textured surfaces. which results in higher maximum profile heights necessary to calculate the MPD (Figure 16). The described phenomena regarding measurements using the volumetric and profilometric methods of EAC pavements influence the values of the b1 coefficients of the linear regression between the MTD and MPD parameters.

Comparative analyzes of the texture of various pavements are based on values recorded mainly on wearing courses of asphalt mixtures. Unlike EAC pavements, BBTM, SMA, PA and AC wearing courses have a negative profile. However, differences in the profile may significantly affect the interpretation of the results. Therefore, measurements on EAC pavements made it possible to verify the relationships between parameters characterizing the texture based on various measurement techniques and to refer to linear regressions developed by other researchers. The planned studies will concern the inclusion of the results obtained in detailed analyses of wearing layers of asphalt mixture types, varying in terms of the content of voids. This will allow us to develop regression relationships that will take into account the technological details necessary for use in practice.

## 5. Conclusions

Texture is crucial to user safety due to the role it plays in generating the friction force between tires and the pavement in slipping conditions. Texture is related to the technology of making the upper road layer. Therefore, at the design stage, it is important to consciously choose individual materials, the type of pavement or texturing technique in the case of concrete pavement.

Based on the analysis of the results of parameters describing microtexture and macrotexture recorded on five test sections with Exposed Aggregate Concrete surface and their comparison with data from the literature, the following conclusions were formulated:

1. Both DFT and BPT devices can be used to estimate the microtexture of EAC pavements. The DFT device is less sensitive to changes occurring in the microtexture compared to BPT. BPN values confirm that the left wheel track is most exposed to polishing factors.
2. Based on the developed linear regression relationship, it was found that DFT20 explains 55% of the values of the independent variable BPN. BPT and DFT20 device measurements allow for the ranking of test areas in terms of microtexture variability.
3. The EAC macrotexture can be verified by volumetric and profilometric methods using the CTM and SLP. The lower MTD values obtained compared to the MPD values are probably related to the positive EAC surface profile. The average difference between MTD and MPD_SLP_ values is approximately 0.34 mm, and between MTD and MPD_CTM_ approximately 0.27 mm.
4. Statistical analysis proved that there is almost certain correlation in the individual configuration of MPD_CTM_, MPD_SLP_ and MTD parameter pairs (correlation coefficient r above 0.90).
5. The deviation from the average MPD value calculated from measurement data on five test sections is approximately 0.40 mm obtained for both the CTM and SLP devices. Slight differences in the results of MPD_CTM_ and MPD_SLP_ are caused by the sampling frequency of the profile scanning laser and the operating characteristics of the devices. However, this does not significantly affect the variability of the obtained results, and CTM and SLP profilometers can be used interchangeably.
6. During comparative analyzes of the results relating to the macrotexture obtained using different techniques, the technology of making the upper layers surface should be taken into account. Due to their characteristics, EAC pavements should be treated independently compared to asphalt pavements when estimating the MTD/ETD parameter. This is particularly important in the practical use of dependencies when controlling the quality of this type of surface.

## Figures and Tables

**Table 1:** Descriptive statistics of the DFT20 and BPN parameters.

	TS−1			TS−2			TS−3			TS−4			TS−5		
	R	C	L	R	C	L	R	C	L	R	C	L	R	C	L
$\bar{DFT20}$ [-]	0.32	0.37	0.32	0.37	0.39	0.35	0.42	0.43	0.42	0.40	0.40	0.38	0.33	0.37	0.33
STD [-]	0.04	0.02	0.02	0.02	0.03	0.02	0.02	0.02	0.02	0.02	0.02	0.03	0.02	0.03	0.02
V [%]	11.8	5.1	5.1	5.2	8.2	4.9	5.3	5.3	4.1	5.4	5.0	6.9	6.8	7.7	5.4
$\bar{BPN}$ [-]	50	55	46	56	58	55	58	61	54	54	57	51	51	54	48
STD [-]	1.73	2.64	2.31	1.39	2.15	2.81	2.60	2.67	2.04	2.23	2.76	2.78	1.29	2.89	1.82
V [%]	3.5	4.8	5.0	2.5	3.7	5.1	4.5	4.3	3.7	4.1	4.8	5.4	2.6	5.3	3.8

**Table 2:** Descriptive statistics of MTD, MPD_CTM_ and MPD_SPL_ parameters on test sections.

	TS−1			TS−2			TS−3			TS−4			TS−5		
	SP	SS	SL	SP	SS	SL	SP	SS	SL	SP	SS	SL	SP	SS	SL
$\bar{MTD}\;$[mm]	1.25	1.29	1.28	0.91	0.81	0.86	0.99	1.05	0.99	1.04	1.12	1.03	0.67	0.66	0.54
STD [mm]	0.27	0.27	0.29	0.06	0.07	0.12	0.08	0.05	0.06	0.10	0.10	0.09	0.07	0.04	0.09
V [%]	21.3	21.1	22.3	7.0	8.2	14.1	7.9	5.1	6.3	9.8	8.5	8.9	11.1	6.2	15.9
$\bar{{MPD}_{CTM}}$ [mm]	1.63	1.69	1.57	1.18	1.10	1.12	1.26	1.33	1.21	1.33	1.39	1.24	0.89	0.83	0.76
STD [mm]	0.26	0.36	0.28	0.08	0.07	0.08	0.08	0.09	0.07	0.09	0.12	0.09	0.13	0.10	0.06
V [%]	16.0	21.1	17.6	6.6	6.1	6.7	6.6	7.0	6.0	6.5	8.9	7.5	14.7	11.6	7.3
$\bar{{MPD}_{SLP}}$ [mm]	1.69	1.81	1.66	1.20	1.21	1.18	1.33	1.40	1.36	1.38	1.52	1.39	0.90	0.91	0.77
STD [mm]	0.32	0.45	0.37	0.07	0.09	0.09	0.07	0.08	0.07	0.16	0.07	0.11	0.10	0.10	0.11
V [%]	19.1	25.0	22.6	6.2	7.7	7.7	5.4	5.8	4.8	11.8	4.9	8.0	11.7	11.1	14.3

**Table 3:** Linear relationships between DFT20 and BPN.

Relationship	R^2^	No. of Reference
DFT20 = 0.016 + 0.0066BPN	0.55	Authors
DFT20 = −0.400 + 0.017BPN	0.86	[34]
DFT20 = 0.018 + 0.0051BPN	0.39	[35]
DFT20 = −0.0668 + 0.0089BPN	0.51	[36]
DFT20 = 0.0517 + 0.0023BPN	0.36	[40]

**Table 4:** Regression relationship between MTD and MPD.

Relationship	R^2^	Measurement Method of MPD	No. of Reference
MTD = 0.76MPD_CTM_	0.87	Circular Texture Meter	Authors
MTD = 0.71MPD_SPL_	0.89	Stationary Laser Profilograph	Authors
ETD = 1.1MPD	-	-	[11], 2019
ETD = 0.2 + 0.8MPD	-	-	[11], 2005
MTD = 0.0762 + 1.2587MPD	0.89	Circular Texture Meter	[15]
MTD = 0.016 + 1.167MPD_ZS_	0.94	ZScanner^®^ 800	[17]
MTD = -0.12 + 1.10MPD_CTM_	0.94	Circular Texture Meter	[19]
MTD = 0.053 + 0.91MPD_CTM_	0.98	Circular Texture Meter	[34]
MTD = 0.069 + 0.947MPD	-	Circular Texture Meter	[39]
MTD = 1.17MPD	0.98	Laser scanner	[41], a
MTD = 0.139 + 0.96MPD	0.91	Laser profiler	[41], b

## Data Availability

The raw data supporting the conclusions of this article will be made available by the authors on request.
